# A Low-Profile High-Gain and Wideband Log-Periodic Meandered Dipole Array Antenna with a Cascaded Multi-Section Artificial Magnetic Conductor Structure

**DOI:** 10.3390/s19204404

**Published:** 2019-10-11

**Authors:** Son Trinh-Van, Oh Heon Kwon, Euntae Jung, Jinwoo Park, Byunggil Yu, Kichul Kim, Jongwoo Seo, Keum Cheol Hwang

**Affiliations:** 1 Department of Electrical and Computer Engineering, Sungkyunkwan University, Suwon 440-746, Korea; jsonbkhn@gmail.com (S.T.-V.); actto389@gmail.com (O.H.K.); 2 Tactical Communication Team, Hanwha Systems, Seongnam 13524, Korea; euntae.jung@hanwha.com (E.J.); jw0822.park@hanwha.com (J.P.); byunggil77.yu@hanwha.com (B.Y.); 3 2nd R&D Institute − 1st Directorate, Agency for Defense Development, Daejeon 34186, Korea; kimkc@add.re.kr (K.K.); jwseo@add.re.kr (J.S.)

**Keywords:** artificial magnetic conductor, log-periodic dipole array (LPDA), low-profile, meandered dipole antenna, wideband

## Abstract

This paper presents a low-profile log-periodic meandered dipole array (LPMDA) antenna with wideband and high gain characteristics. The antenna consists of 14 dipole elements. For compactness, a meander line structure is applied to each dipole element to reduce its physical length. As a result, a compact and wideband LPMDA antenna is realized, exhibiting a wide impedance bandwidth of 1.04–5.22 GHz (ratio bandwidth of 5.02:1) for |$S_{11}$| < −10 dB. To enhance the antenna gain performance while maintaining the wideband behavior, the LPMDA antenna is integrated with a new design of an artificial magnetic conductor (AMC) structure. The designed AMC is realized by combining three AMC structures of different sizes to form a cascaded multi-section AMC structure, of which its overall operating bandwidth can continuously cover the entire impedance bandwidth of the LPMDA antenna. The proposed AMC-backed LPMDA antenna is experimentally verified and its measured −10 dB reflection bandwidth is found to be in the range of 0.84–5.15 GHz (6.13:1). At the main beam direction within the operating frequency bandwidth, the gain of the proposed AMC-backed LPMDA antenna ranges from 7.15–11.43 dBi, which is approximately 4 dBi higher than that of an LPMDA antenna without an AMC. Moreover, the proposed antenna has a low profile of only 0.138$\lambda_{L}$. ($\lambda_{L}$ is the free-space wavelength at the lowest operating frequency).

## 1. Introduction

The rapid development of modern wireless communication systems has increased the demand for wideband and high-gain antennas [1,2,3,4]. The log-periodic dipole array (LPDA) antenna, as a type of frequency-independent antenna, has been a popular choice in communication systems since the pioneering work of Isbell [5] due to its advantages of a wideband, high gain, and a simple antenna design. However, one of the problems with a conventional LPDA antenna is its typically large size, as a conventional LPDA antenna is basically an array of λ/2-dipole elements, and its transverse size is determined by the length of the longest element, which is equal to $\lambda_{L}/2$ (with $\lambda_{L}$ being the free-space wavelength at the lowest operating frequency). Therefore, the size of an LPDA antenna should be minimized to make it more applicable to various communication applications in which the mandated space for an antenna is restricted. For miniaturization of an LPDA antenna, several techniques have been introduced [6,7,8,9,10,11]. In one study [6], a Koch-fractal configuration was implemented in an LPDA antenna, resulting in a size reduction of 18% as compared to a conventional LPDA antenna. A LPDA miniaturized by implementing cylindrical-hat covers on dipole elements was presented and a 31% reduction in the length of the longest dipole element was realized [7]. In other studies, a compact dielectric-loaded LPDA antenna [8] and a T-shaped top-loaded LPDA antenna [9] were introduced, achieving length reductions of 44% and 45%, respectively. Miniaturization using meandered dipole elements was also investigated to reduce the sizes of LPDA antennas [10,11].

More recently, it has been found to be a challenge for antenna engineers to design low-profile and high-gain antennas with wideband characteristics for communication systems used in aircrafts, missiles, and unmanned aerial vehicles (UAVs), in which the antennas are usually flush-mounted on a large metallic platform while the antenna profile is highly restricted in the vertical direction owing to aerodynamic and mechanical requirements. There are only a few types of antennas that are promising for such applications. An H-plane ridged substrate-integrated waveguide (SIW) horn antenna mounted on a large ground plane was introduced [12]. This antenna provided a wide bandwidth of 2.73:1 with a VSWR of less than 2.5 by employing an arc-shaped horn aperture loaded with a dielectric material and a wideband coaxial probe to three-step ridged SIW transition. Nevertheless, this antenna structure is somewhat complicated, which makes it difficult to fabricate, especially the cone-shaped feeding pin inside the SIW structure and the three-step ridge. In another work [13], a flush-mounted surface-wave antenna with an overall height of 0.127$\lambda_{L}$ was designed, reportedly showing a ratio bandwidth of 4:1 with a VSWR of less than 2. However, its transverse dimension is very large (approximately 1.54$\lambda_{L}$), resulting in a bulky structure. Recently, a low-profile surface-wave antenna was realized by employing a grounded ceramic slab with a very high dielectric constant ($\varepsilon_{r}$ = 25) [14]. However, this antenna achieved a ratio bandwidth of only 2.98:1. Taking advantage of the very wide bandwidth characteristic, log-periodic array antennas are also suitable for mounting on a large metallic platform. In one study [15], a low-profile log-periodic monopole array antenna with a quasi-planar structure was introduced. A top-hat loading technique was applied, greatly reducing the height of the monopole elements to 0.047$\lambda_{L}$. This antenna exhibited a ratio bandwidth of 4.53:1 with a VSWR of less than 2.3. An ameliorated design of the original antenna [15] was also reported [16], showing a very low profile of only 0.053$\lambda_{L}$ and a wide ratio bandwidth of 9.15:1 with a VSWR of less than 2. However, this antenna structure is somewhat complicated, requiring a very precise process to implement vertical monopoles of different heights onto the feeding network. In another work [17], a low-profile log-periodic microstrip patch antenna backed with an artificial magnetic conductor (AMC) surface was reported, achieving a narrow ratio bandwidth of only 1.51:1. The AMC surface is a periodic structure consisting of a two-dimensional array of tightly coupled unit cells of square metallic patches [18]. Similar to a perfect magnetic conductor (PMC), when placed under an antenna, AMC surface exhibits in-phase reflection characteristics within a specific frequency band. Therefore, the AMC surface can operate as a reflector when located closer to the antenna than a general perfect electric conductor (PEC) reflector would be [19,20], forming a low-profile AMC-backed antenna design.

In this paper, we propose a low-profile, high-gain, and wideband log-periodic meandered dipole array (LPMDA) antenna with an AMC backing. First, the dipole elements are designed using the meandering technique and are optimized based on a binary genetic algorithm (GA) [21,22,23,24] to realize a compact and wideband LPMDA antenna. Second, a novel cascaded multi-section AMC structure designed to exhibit a wide in-phase reflection bandwidth covering the entire operating bandwidth of the LPMDA is developed and applied as a reflector for the LPMDA antenna to enhance its gain. Compared to previous work [11], the proposed cascaded multi-section AMC structure enhances the in-phase reflection bandwidth by approximately two and half times by cascading three AMC sections of difference sizes. Our proposed AMC-backed LPMDA antenna has a low profile of 0.138$\lambda_{L}$, a very wide ratio bandwidth of 6.13:1 for |$S_{11}$| < −10 dB, and a high gain exceeding 7.15 dBi over the entire operating frequency band, demonstrating its potential to be useful for aircrafts, missile, and UAVs. In this study, the commercially available software ANSYS high-frequency structure simulator (HFSS) is used to conduct all of the simulations. The following sections describe in detail the antenna geometry and discuss the experimental results to verify the feasibility of the proposed antenna.

## 2. Antenna Design and Experimental Results

### 2.1. Log-Periodic Meandered Dipole Array (LPMDA) Antenna Design

**Figure 1:**
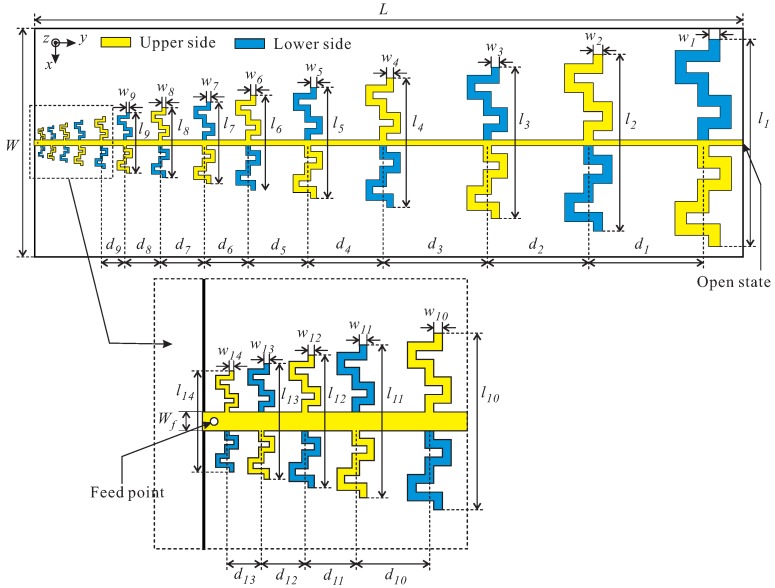
Configuration of the log-periodic meandered dipole array (LPMDA) antenna.

**Figure 2:**
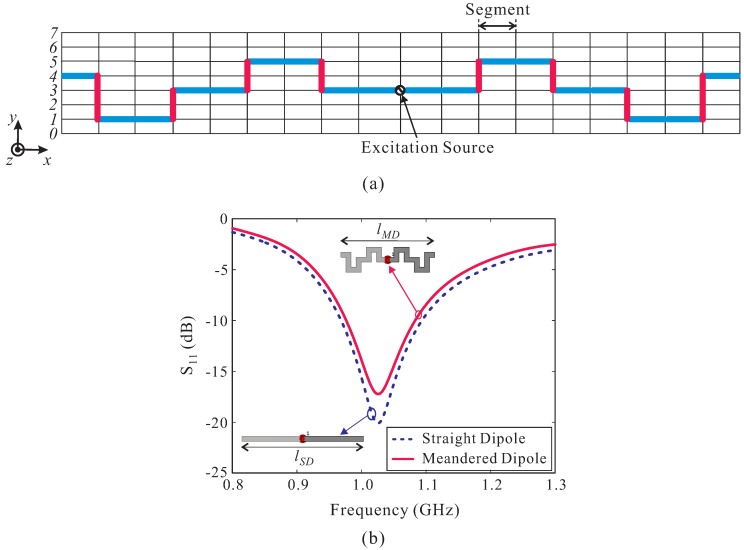
(**a**) Design concept of the meandered dipole element; (**b**) Simulated |$S_{11}$| of the meandered dipole antenna and a straight dipole antenna. ($l_{MD}$ = 90.65 mm and $l_{SD}$ = 115.50 mm).

Figure 1 shows the configuration of the log-periodic meandered dipole array (LPMDA) antenna. The substrate used to design antenna in this work is a Taconic RF-35 substrate with a thickness of 1.52 mm, a dielectric constant ($\varepsilon_{r}$) of 3.5, and a loss tangent (tanδ) of 0.0019. The conductors on the upper and lower sides are indicated in yellow and blue, respectively. The feeding point is located at one end of the double-sized parallel transmission strip line near the shortest element. The other end of the double-sized parallel transmission strip line simply has an open circuit; no load is needed at the far end. Here, *W*, *L*, and $w_{f}$ denote the width and length of the antenna substrate and the width of the parallel transmission strip line, respectively. In this design, the LPMDA antenna is composed of fourteen dipole elements. The dipole element is designed using the meandering technique for miniaturization. The design concept of the meandered dipole antenna, recently developed by Bayrakatar et al. in their effort to design a miniaturized three-element Yagi-Uda array [25], is illustrated in Figure 2a. The design process starts by dividing each arm of the dipole element into nine segments of equal lengths (as denoted in blue). These segments are then put into a fixed 8 × 18 grid. Each segment lies on one column at one of the positions numbered ‘0’ through ‘7’ (in order from the bottom; see Figure 2a). A meandered line configuration is formed by connecting two segments on the two adjacent columns through a vertical line (as denoted in red). The optimal position of each segment in the corresponding column is selected through a binary GA optimization process, resulting in a meandered line scheme which performs comparably the conventional straight line configuration. To verify whether the meandered line scheme can effectively reduce the length, a meandered dipole antenna and a conventional straight dipole antenna are investigated, as shown in the insets of Figure 2b. The simulated reflection coefficients of the two antennas are plotted in Figure 2b. As observed, the two dipole antennas resonate at the same frequency of approximately 1.03 GHz. To realize the resonance at this frequency, the length, $l_{SD}$, of the conventional straight dipole is 115.50 mm. Meanwhile, for the meandered dipole antenna, its length $l_{MD}$ is only 90.65 mm. Therefore, a length reduction of approximately 21.5% is achieved by applying the meandering technique.

**Figure 3:**
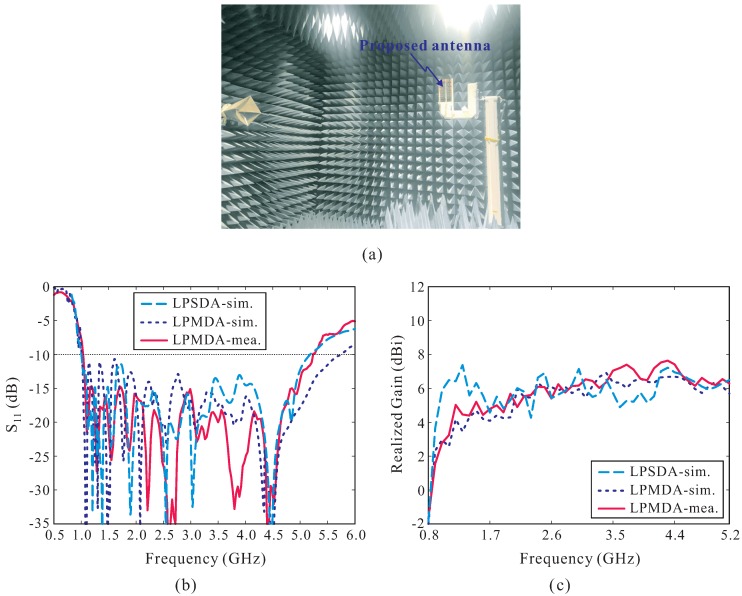
(**a**) Measurement setup for the far-field radiation pattern in the anechoic chamber; (**b**) Reflection coefficients; (**c**) Realized Gains.

The meander line configuration is then applied to design a miniaturized 14-element LPMDA antenna, in which the aforementioned meandered dipole element is used as the longest dipole element (as shown in Figure 1). It is important to note that the transverse size of a log-periodic dipole antenna is determined by the length of its longest element. Therefore, it can be expected that the designed LPMDA antenna also can achieve a 21.5% size reduction when compared to its conventional counterpart. The length $l_{n}$ and width $w_{n}$ of each dipole element can be determined based on the Carrel method [26],

(1) $$\tau =\frac{l_{n+1}}{l_{n}}=\frac{w_{n+1}}{w_{n}}$$

where *n* is the series number (*n* = 1, 2…, 14) and τ is the scaling factor. Here, the length $l_{1}$ and the width $w_{1}$ of the longest element are fixed at 90.65 mm and 4.90 mm, respectively. To achieve a good impedance matching over a broad frequency range while maintaining the compactness of the LPMDA antenna, several major design parameters, in this case the scaling factor τ and element spacings from $d_{1}$ to $d_{13}$, are selected for inclusion in the binary GA optimization process. During the implementation of the binary GA optimization, each of these design parameters is encoded into eight bits. For each segment, three additional bits are used to realize eight states, each of these states corresponds to one position on each column of the grid (see Figure 2a). Therefore, in total, a 139-bit binary string is utilized to perform binary GA optimization. Binary GA optimization is developed with MATLAB programming linked to HFSS through an HFSS scripting interface and is implemented with one hundred iterations, a population of 20, a mutation rate of 0.15, and the single-point crossover scheme [21]. In the HFSS simulation, each segment of the longest dipole element is modeled by a rectangular patch 4.90 × 4.90 mm${}^{2}$ in size. The optimized design parameters of the LPMDA are summarized in Table 1. A prototype of the LPMDA antenna with overall dimensions of 300 × 100 × 1.52 mm${}^{3}$ is fabricated and tested. An Agilent 8510C network analyzer was used to measure the reflection coefficients. The far-field radiation pattern was measured in an anechoic chamber 5.5 m × 5.5 m × 5 m in size. The radiation pattern measurement setup is shown in Figure 3a. The simulated and measured results of the reflection coefficients and realized gains of the LPMDA antenna are illustrated in Figure 3b,c. The simulated results of the reflection coefficient and realized gain of the log-periodic straight dipole array (LPSDA) antenna are also included in Figure 3b,c for comparison, respectively. Note that the LPSDA is designed based on the longest element, with a length of 115.50 mm. As observed in Figure 3b, the lowest operating frequencies of the LPSDA and LPMDA are nearly identical, indicating that a transverse length reduction is achieved for the LPMDA. The simulated and measured −10 dB reflection bandwidths of the LPMDA are 1.01–5.74 GHz (5.68:1) and 1.04–5.22 GHz (5.02:1), respectively. As shown in Figure 3c, when applying the meandering technique to achieve a size reduction of 21.5%, the gain of the LPMDA antenna in the lower frequency range is reduced by nearly 2 dBi as compared to that of the LPSDA antenna. We consider this gain reduction compared to the reduced antenna size to be an acceptable trade-off. Moreover, degradation of the gain can be compensated when integrating the LPMDA with the AMC structure as a reflector. This will be presented and discussed in detail in the following sections. For the LPMDA, within the operating frequency band, the measured realized gains in the direction of the main beam range from 2.78 to 7.64 dBi (see Figure 3c). A comparison of some key features is conducted between the presented LPMDA antenna and other miniaturized log-periodic array antennas described in the literature [7,9,10,11], as shown in Table 2. Clearly, the LPMDA antenna presented here has a much wider ratio bandwidth and a higher gain, although it occupies a slightly larger space.

### 2.2. Design of the Cascaded Multi-Section AMC Structure

**Figure 4:**
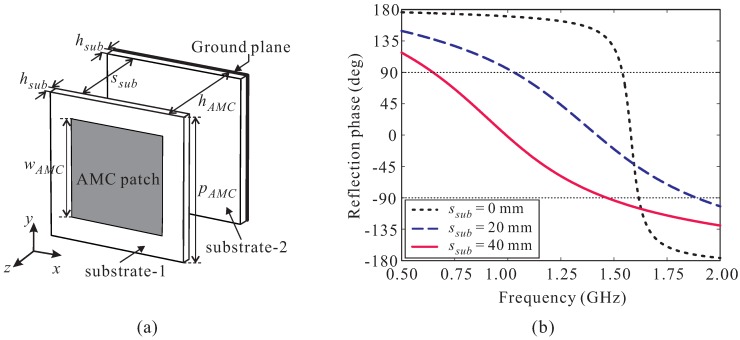
(**a**) Geometry of the artificial magnetic conductor (AMC) unit cell; (**b**) Simulated reflection phase versus the spacing between two substrates.

In this section, we present a new design of an AMC structure with very wide bandwidth characteristics capable of covering the entire operating bandwidth of the LPMDA antenna from 1.04 to 5.22 GHz. Normally, the conventional AMC structure exhibits a limited in-phase reflection bandwidth around a specific frequency. As demonstrated in the literature [11], the in-phase reflection bandwidth can be enhanced significantly with the AMC structure is created on two separated substrates. In this design approach, the square metallic patch of the AMC is printed on the top layer of the upper substrate (substrate-1) and the ground plane is on the bottom layer of the lower substrate (substrate-2), as shown in Figure 4a. Both substrates are the Taconic RF-35 types with a thickness of $h_{sub}$ = 1.52 mm. The two substrates are separated by a spacing of $s_{sub}$. The overall height $h_{AMC}$ of the AMC structure is calculated as $h_{AMC}$ = $s_{sub}$ + 2$h_{sub}$.

**Figure 5:**
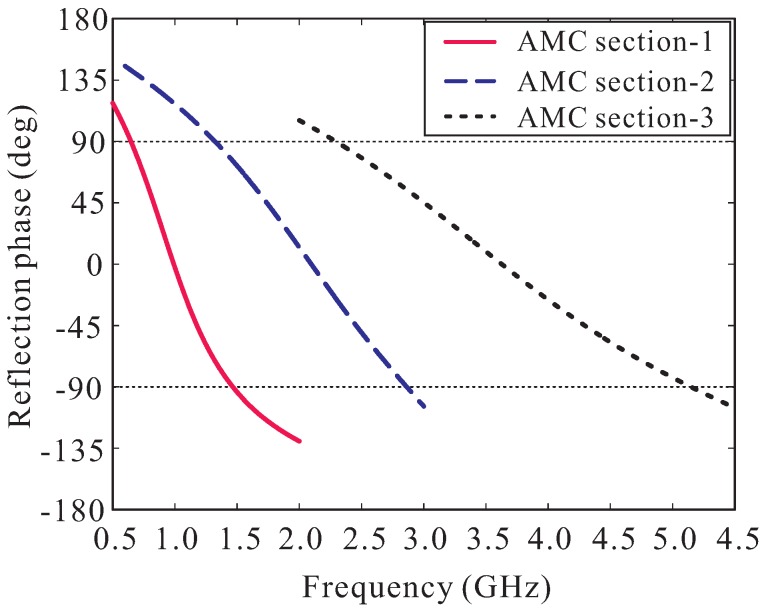
Simulated reflection phase of the proposed cascaded multi-section AMC structure. AMC section-1: $p_{AMC1}$ = 70 mm, $w_{AMC1}$ = 48 mm, $h_{AMC1}$ = 43.04 mm. AMC section-2: $p_{AMC2}$ = 35 mm, $w_{AMC2}$ = 21 mm, $h_{AMC2}$ = 23.04 mm. AMC section-3: $p_{AMC3}$ = 35 mm, $w_{AMC3}$ = 14 mm, $h_{AMC3}$ = 13.04 mm.

A full-wave simulation is conducted to investigate the performance of the AMC structure in terms of its reflection phase properties. Two PEC walls and two PMC walls are used to simulate in infinite-sized periodical structure [19,20]. The useful bandwidth of the AMC, also referred to as in-phase reflection bandwidth, is generally defined from the frequency point at +90${}^{{}^{\circ}}$ to that at −90${}^{{}^{\circ}}$. Figure 4b illustrates the simulated reflection phase of the AMC structure versus the spacing $s_{sub}$. For $s_{sub}$ = 0 mm, the AMC exhibits a very narrow in-phase reflection bandwidth of only 1.54–1.61 GHz. When increasing $s_{sub}$, the reflection phase curve shifts to a lower frequency range and its curve slope is reduced significantly, enhancing the in-phase reflection bandwidth of the AMC. At $s_{sub}$ = 40 mm, the in-phase reflection bandwidth of the AMC is 0.64–1.47 GHz. However, this bandwidth does not sufficiently support the designed LPMDA antenna. Meanwhile, with $s_{sub}$ = 40 mm, the total height, $h_{AMC}$, of the AMC is 43.04 mm, which is approximately 0.087$\lambda_{at\;0.64\;GHz}$. Increasing the spacing $s_{sub}$ further to broaden the in-phase reflection bandwidth is not the best solution due to the requirement of a low-profile design. Instead, we apply a scaling technique to this AMC structure. Two additional down-scaled AMCs are realized to exhibit in-phase reflection bandwidths at two different frequency ranges. The parameters of these AMCs are then carefully tuned so that three in-phase reflection bandwidths of three AMCs are partially overlapped to realize a broad operating bandwidth continuously covering the frequency range of 1.04–5.22 GHz. The three AMCs are then cascaded to form a cascaded multi-section AMC structure. The simulated reflection phase property of the proposed cascaded multi-section AMC structure is shown in Figure 5 and the parameters for each AMC section are listed in the caption. As observed, AMC section-1 has an in-phase reflection bandwidth of 0.64–1.47 GHz. For AMC section-2 and AMC section-3, their in-phase reflection bandwidths are 1.32–2.86 GHz and 2.28–5.24 GHz, respectively. Therefore, the proposed cascaded multi-section AMC structure provides a very broad in-phase reflection bandwidth of 0.64–5.24 GHz, entirely covering the operating bandwidth of the LPMDA antenna, while still keeping the overall AMC height low at 0.087$\lambda_{at\;0.64\;GHz}$.

### 2.3. The Proposed AMC-Backed LPMDA Antenna

**Figure 6:**
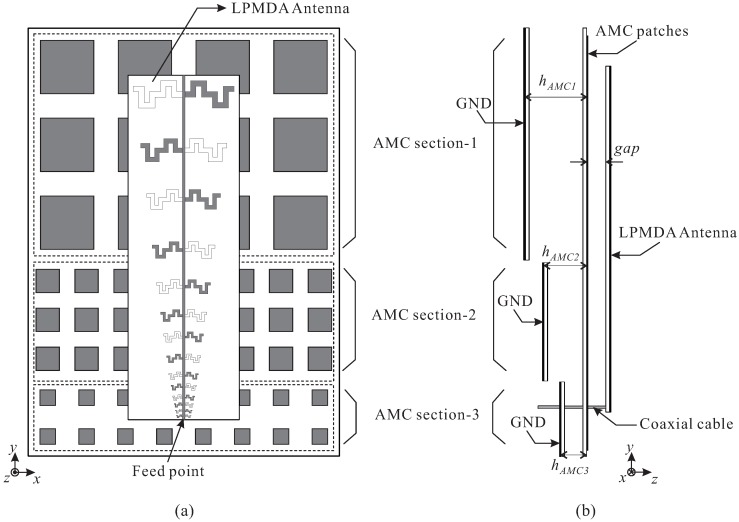
Geometry of the proposed AMC-backed LPMDA antenna: (**a**) Top-view; (**b**) Side-view.

The LPMDA antenna and the cascaded multi-section AMC structure are combined to realize the proposed low-profile AMC-backed LPMDA antenna, as displayed in Figure 6. For simplicity, all of the AMC patches of the three AMC sections are printed on the top layer of a single Taconic RF-35 substrate. Three ground planes are separated and positioned at distances corresponding to the heights $h_{AMC}$ of the AMC sections. The LPMDA antenna is placed above the AMC structure at a distance of gap = 5 mm. AMC section-1, consisting of 3 × 4 unit cells, is used to support the first four dipole elements. The next five dipole elements are covered by the 3 × 8 AMC section-2, and the last five dipole elements are supported by the 2 × 8 AMC section-3. The proposed antenna is fed by a coaxial cable.

**Figure 7:**
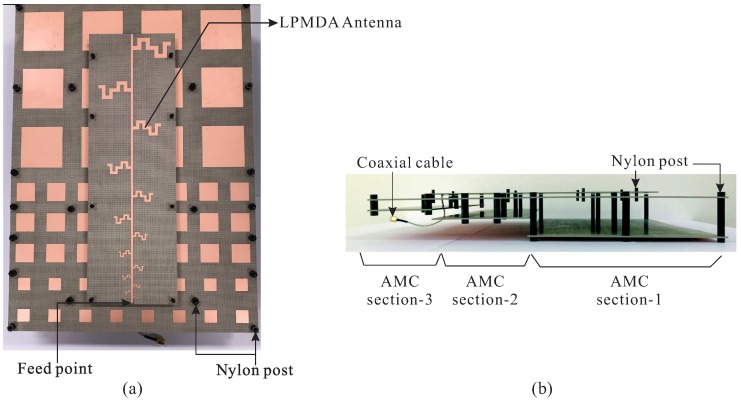
Photograph of the fabricated antenna: (**a**) Top-view; (**b**) Side-view.

**Figure 8:**
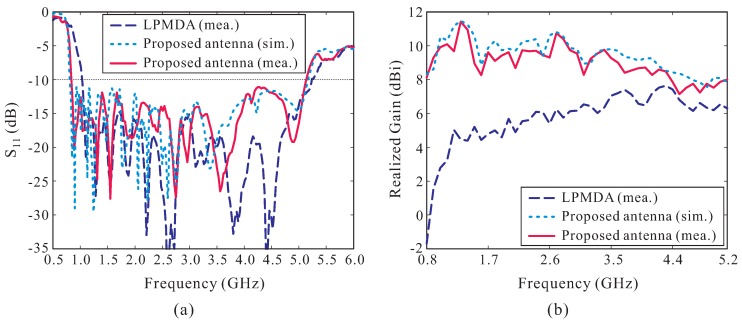
Performance of the proposed antenna: (**a**) Reflection coefficients; (**b**) Realized Gains.

A prototype of the proposed AMC-backed LPMDA antenna is fabricated and experimentally verified. Figure 7 shows a photograph of the fabricated antenna. Nylon posts are utilized to support the LPMDA antenna above the AMC and to create air gaps between the substrates that constitute the cascaded multi-section AMC structure. The overall dimensions of the fabricated antenna are 385 × 280 × 49.56 mm${}^{3}$. Figure 8 presents the simulated and measured reflection coefficients and realized gains at the main beam direction of the proposed AMC-backed LPMDA antenna. The measurement is found to be in good agreement with those from the simulation. The discrepancy can be attributed to inaccuracies during the fabrication process, experimental tolerances, and to the assembly process. The measured reflection coefficient and realized gain of the LPMDA antenna without the AMC are also shown here for comparison. Under the condition of |$S_{11}$| < −10 dB, the measured and simulated operating bandwidths of the proposed AMC-backed LPMDA antenna are 0.84–5.15 GHz (6.13:1) and 0.81–5.17 GHz (6.38:1), respectively (see Figure 8a). Compared to the LPMDA antenna without the AMC, the lowest operating frequency is reduced from 1.04 to 0.84 GHz by applying the AMC. At the lowest operating frequency of 0.84 GHz, the proposed antenna has a height of only 0.138$\lambda_{L}$ and is thus a low-profile antenna design. As observed in Figure 8b, when the AMC is applied to the proposed antenna, realized gain at the main beam direction is enhanced significantly at the lower frequency range as compared to the LPMDA antenna without the AMC. The measured realized gains of the proposed AMC-backed LPMDA antenna range from 7.15 to 11.43 dBi within the operating frequency band.

**Figure 9:**
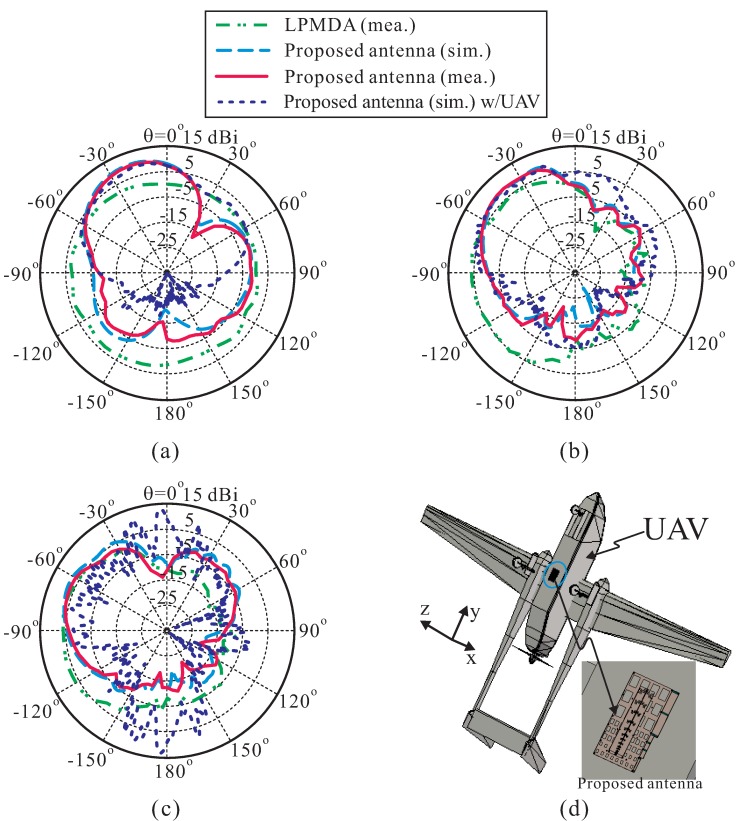
Simulated and measured radiation patterns of the proposed AMC-backed LPMDA antenna on the *yz*-plane: (**a**) at 1.0 GHz; (**b**) at 3.0 GHz; (**c**) at 4.5 GHz; (**d**) Simulation model of the proposed AMC-backed LPMDA antenna mounted below the unmanned aerial vehicle (UAV) platform.

Figure 9a–c illustrate the simulated and measured radiation patterns on the *yz*-plane of the proposed antenna at three different frequencies of 1.0 GHz, 3.0 GHz, and 4.5 GHz. Good agreement is also achieved between the simulation and measurement. Again, the measured radiation patterns of the LPMDA antenna without the AMC are included for comparison. Clearly, the LPMDA antenna exhibits end-fire radiation; the main beam direction is formed at θ = −90${}^{{}^{\circ}}$. However, the main beam of the proposed AMC-backed LPMDA antenna deviates from the end-fire direction by 70${}^{{}^{\circ}}$ (at 1 GHz), 45${}^{{}^{\circ}}$ (at 3 GHz), and 25${}^{{}^{\circ}}$ (at 4.5 GHz) because of the effect of the AMC, which acts as a reflector. It is important to note that the main beam can be closer to the end-fire direction when the proposed antenna is mounted on a very large ground plane [14].

To assess the practicality of the proposed design with regard to its application to an unmanned aerial vehicle (UAV), a simulation of the proposed AMC-backed LPMDA antenna mounted on a simplified version of a UAV is conducted. The UAV in this case consists of a fuselage, twin tailbooms, and two wings, with all surfaces implemented as conductors. In the simulation, the proposed antenna is located below the center of the UAV’s fuselage, as shown in Figure 9d. Simulated far-field radiation patterns of the proposed antenna mounted on the UAV in *yz*-plane (pitch plane) are also shown in Figure 9a–c for frequencies of 1.0 GHz, 3.0 GHz, and 4.5 GHz, respectively. It can be seen that the radiation patterns are slightly influenced by the UAV structure at lower frequencies. However, at higher frequencies, the effects of the fuselage and tailbooms are distinctly manifested on the pitch plane, as observed from Figure 9c.

Table 3 summarizes a comparison of the key features between existing antennas described in the literature and our design [14,15,16]. compared to most of previous antennas [14,15], the proposed antenna has a compact size, a compatible gain, and particularly a much wider operating bandwidth. Compared with earlier work [16], the proposed antenna exhibits a higher peak gain with fewer elements.

## 3. Conclusions

A low-profile, high gain, and wideband AMC-backed log-periodic meandered dipole array (LPMDA) antenna was presented, fabricated, and tested. All antenna components were designed using a Taconic RF-35 dielectric substrate. A meander line configuration was applied to the dipole elements to realize a miniaturized LPMDA antenna. To enhance the antenna gain while maintaining the wideband characteristics, a new AMC structure, termed a cascaded multi-section AMC structure, was also developed and integrated with the LPMDA antenna. The experimental results demonstrated that the LPMDA antenna without the AMC and the proposed AMC-backed LPMDA antenna have −10 dB reflection bandwidths of 1.04–5.22 GHz (5.02:1) and 0.84–5.15 GHz (6.13:1), respectively. Within the operating frequency band, the measured gains at the main beam direction range from 2.78 to 7.64 dBi for the LPMDA antenna without the AMC structure and from 7.15 to 11.43 dBi for the proposed AMC-backed LPMDA antenna. In addition, the proposed AMC-backed LPMDA antenna has a low profile of only 0.138$\lambda_{L}$, making it suitable for the communication systems used in UAVs and aircrafts.

## Figures and Tables

**Table 1:** Optimized Geometrical Parameters of the LPMDA Antenna (Units: millimeters).

Parameter	Value	Parameter	Value	Parameter	Value	Parameter	Value
*W*	100	$w_{9}$	1.335	$l_{6}$	41.58	$d_{3}$	45.57
*L*	300	$w_{10}$	1.135	$l_{7}$	35.71	$d_{4}$	32.98
$W_{f}$	2.45	$w_{11}$	0.965	$l_{8}$	30.72	$d_{5}$	26.04
$w_{1}$	4.90	$w_{12}$	0.820	$l_{9}$	26.48	$d_{6}$	19.21
$w_{2}$	4.165	$w_{13}$	0.697	$l_{10}$	22.88	$d_{7}$	19.26
$w_{3}$	3.540	$w_{14}$	0.592	$l_{11}$	19.81	$d_{8}$	15.55
$w_{4}$	3.009	$l_{1}$	90.65	$l_{12}$	17.21	$d_{9}$	10.21
$w_{5}$	2.558	$l_{2}$	77.42	$l_{13}$	15.00	$d_{10}$	9.55
$w_{6}$	2.174	$l_{3}$	66.17	$l_{14}$	13.12	$d_{11}$	6.59
$w_{7}$	1.848	$l_{4}$	56.62	$d_{1}$	50.24	$d_{12}$	5.65
$w_{8}$	1.571	$l_{5}$	48.49	$d_{2}$	44.26	$d_{13}$	4.45

**Table 2:** Performance Comparison with Previous Miniaturized Log-Periodic Array Antennas.

Ref.	Ratio Bandwidth	Peak Gain (dBi)	Overall Dimensions *W* × *L* ($\lambda_{L}^{2}$)	3 dB Gain Bandwidth (%)
[7]	1.58:1	6.2	0.39 × 0.35	44.83
[9]	2.55:1	6.4	0.24 × 0.44	87.29
[10]	4.15:1	6.69	Not Given	Not Given
[11]	2.34:1	7.17	0.37 × 0.62	83.87
This work	5.02:1	7.64	0.31 × 1.04	88.88

The transverse size is determined by the length of the longest element. $\lambda_{L}$ is the free-space wavelength at the lowest operating frequency.

**Table 3:** Performance Comparison with Previous Wideband Low-Profile Antennas.

Ref.	Ratio Bandwidth	Realized Gain (dBi)	Number of Elements	Dimensions ($\lambda_{L}^{3}$)
[14]	2.98:1	4–12	N.A.	1.686 × 0.611 × 0.065
[15] *	4.53:1	4.5–10	15	2.1 × 1.2 × 0.047
[16]	9.15:1	7.2–9.2	21	1.93 × 0.67 × 0.053
This work	6.13:1	7.15–11.43	14	1.078 × 0.784 × 0.138

* The operating bandwidth of this design is defined under the condition of VSWR < 2.3. VSWR: Voltage Standing Wave Ratio.
